# Proteomic dataset of *Listeria monocytogenes* exposed to sublethal concentrations of free and nanoencapsulated nisin

**DOI:** 10.1016/j.dib.2022.108343

**Published:** 2022-05-31

**Authors:** Cristian Mauricio Barreto Pinilla, Paolo Stincone, Adriano Brandelli

**Affiliations:** a Centro de Tecnologia de Lacticínios (TECNOLAT), Instituto de Tecnologia de Alimentos, Campinas, SP 13070-178, Brazil; b CMFI Cluster of Excellence, Interfaculty Institute of Microbiology and Medicine, University of Tübingen, Tübingen 72076, Germany; c Laboratório de Bioquímica e Microbiologia Aplicada, Departamento de Ciência de Alimentos, Universidade Federal do Rio Grande do Sul, Porto Alegre 91501-970, Brazil

**Keywords:** Proteome, Tandem mass spectroscopy, Bacteria, protein analysis, Antimicrobial, Nisin

## Abstract

The cellular proteins of *L. monocytogenes* exposed to free and liposome-encapsulated nisin at sublethal concentration were hydrolyzed by trypsin and examined by tandem mass spectrometry (MS/MS) to obtain proteomic data. In the present study, we use the STRING v11.05 database analyze the interactions among the 78 upregulated proteins from *L. monocytogenes* obtained after treatment with sublethal concentrations of free and nanoliposome-encapsulated nisin. As result, from the upregulated proteins by free nisin was determined a network with 140 edges with two relevant nodes, containing ribosomal proteins and transmembrane transport proteins (SecD and ABC transport system). These two sets of proteins present biological connection as a group, with strong interactions and are related to detoxification and other *Listeria* response mechanisms. In addition, a high amount of membrane proteins was identified in the free nisin treatment. On the other hand, in the interaction analysis of upregulated proteins by liposome-loaded nisin, was found 156 edges with a single protein network, the same observed in free nisin, related to ribosomal proteins. Therefore, according with this analysis, the encapsulation of nisin into liposomes cause upregulation of ribosomal and decrease of *L. monocytogenes* response proteins as compared with free nisin.

## Specifications Table

**Table:** 

Subject	Biological Sciences: Omics: Proteomics
Specific subject area	Proteomics data of *Listeria monocytogenes*
Type of data	Tables and figures
How the data were acquired	Liquid chromatography-tandem mass spectrometric (LC-MS/MS) analysis, using a LTQ Orbitrap Velos mass spectrometer connected to the EASY-nLC system through a Proxeon nanoelectrospray ion source
Data format	Raw data and analyzed
Description of data collection	LC-MS/MS based proteomic profiling of total protein of *Listeria* cells after three treatments: sublethal concentration of free nisin, sublethal concentration of nisin encapsulated in nanoliposomes and unloaded liposomes
Data source location	Institution: Universidade Federal do Rio Grande do Sul City/Town/Region: Porto Alegre/RS Country: Brazil
Data accessibility	Repository name: MassIVE Data identification number: MSV000089076 Direct URL to data: https://massive.ucsd.edu/ProteoSAFe/dataset.jsp?task=451961119585408bacabbc136f28d8fb
Related research article	C.M.B. Pinilla, P. Stincone, A. Brandelli, Proteomic analysis reveals differential responses of *Listeria monocytogenes* to free and nanoencapsulated nisin, Int. J. Food Microbiol. 346 (2021) 109170. doi:10.1016/j.ijfoodmicro.2021.109170

## Value of the Data

• This dataset contains unique information on proteome of *L. monocytogenes* exposed to nanostructured antimicrobial peptide nisin.
• The data may be valuable for scientists of different fields, including microbiology, protein science, food science, and nanotechnology.
• The data can be useful to understand the effect of natural antimicrobials on pathogenic bacteria at molecular level.
• The analysis of data may be used for development of innovative strategies to combat pathogenic bacteria.

## 1 Data Description

**Fig. 1:**
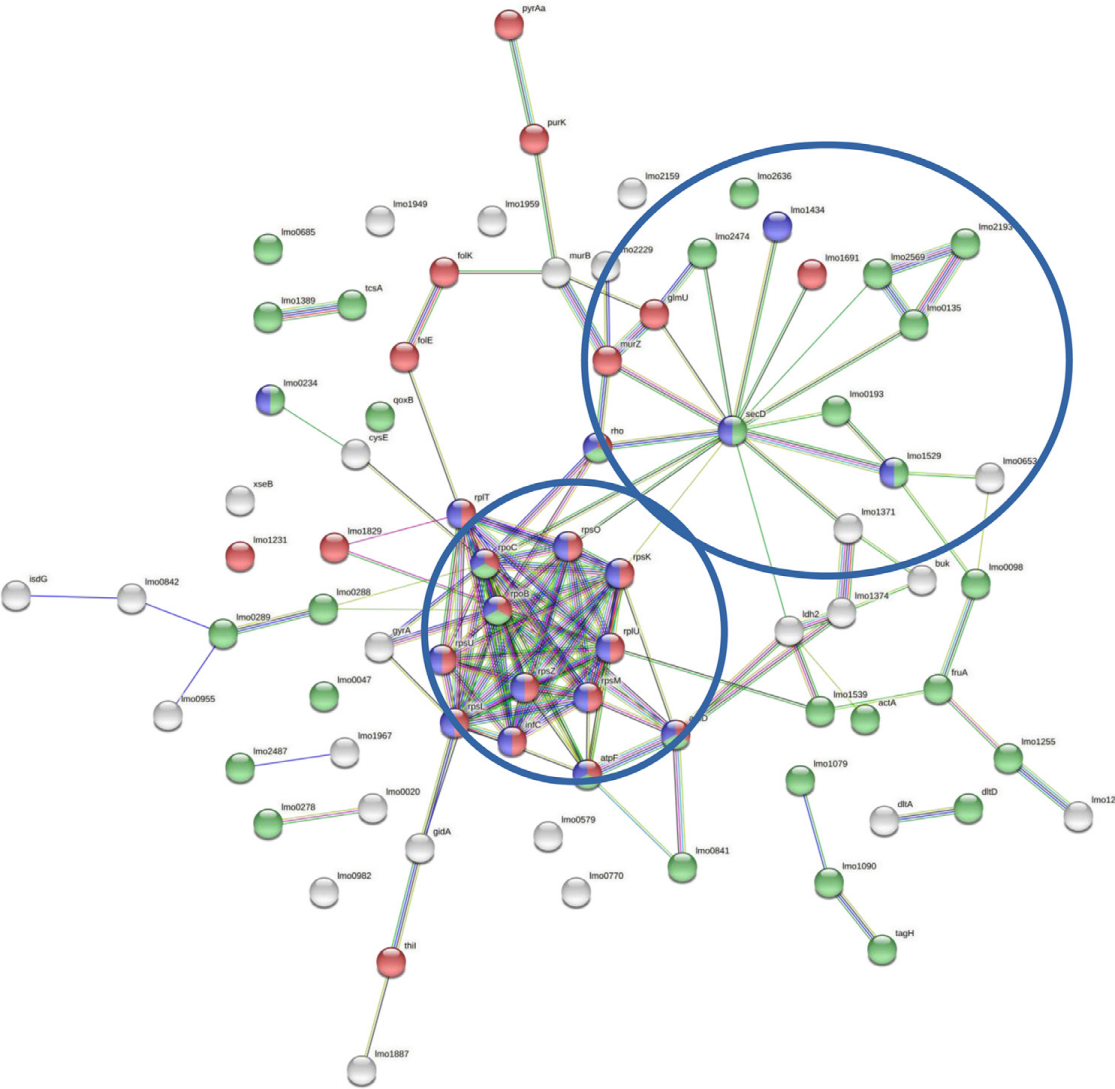
Protein-protein interaction network of upregulated *Listeria monocytogenes* ATCC 7466 proteins after interaction with free nisin. The proteins are represented by nodes whereas their interactions by edges. The line colors indicate different types of know (pink and light blue), predicted (green, red and blue) and other (yellow, black and gray) interactions. The proteins (identified by its code gene) in red color related cellular nitrogen compounds biosynthesis, blue color proteins related to translation and channel activity, and green color with membrane proteins. The network was constructed with STRING v11.05.

**Fig. 2:**
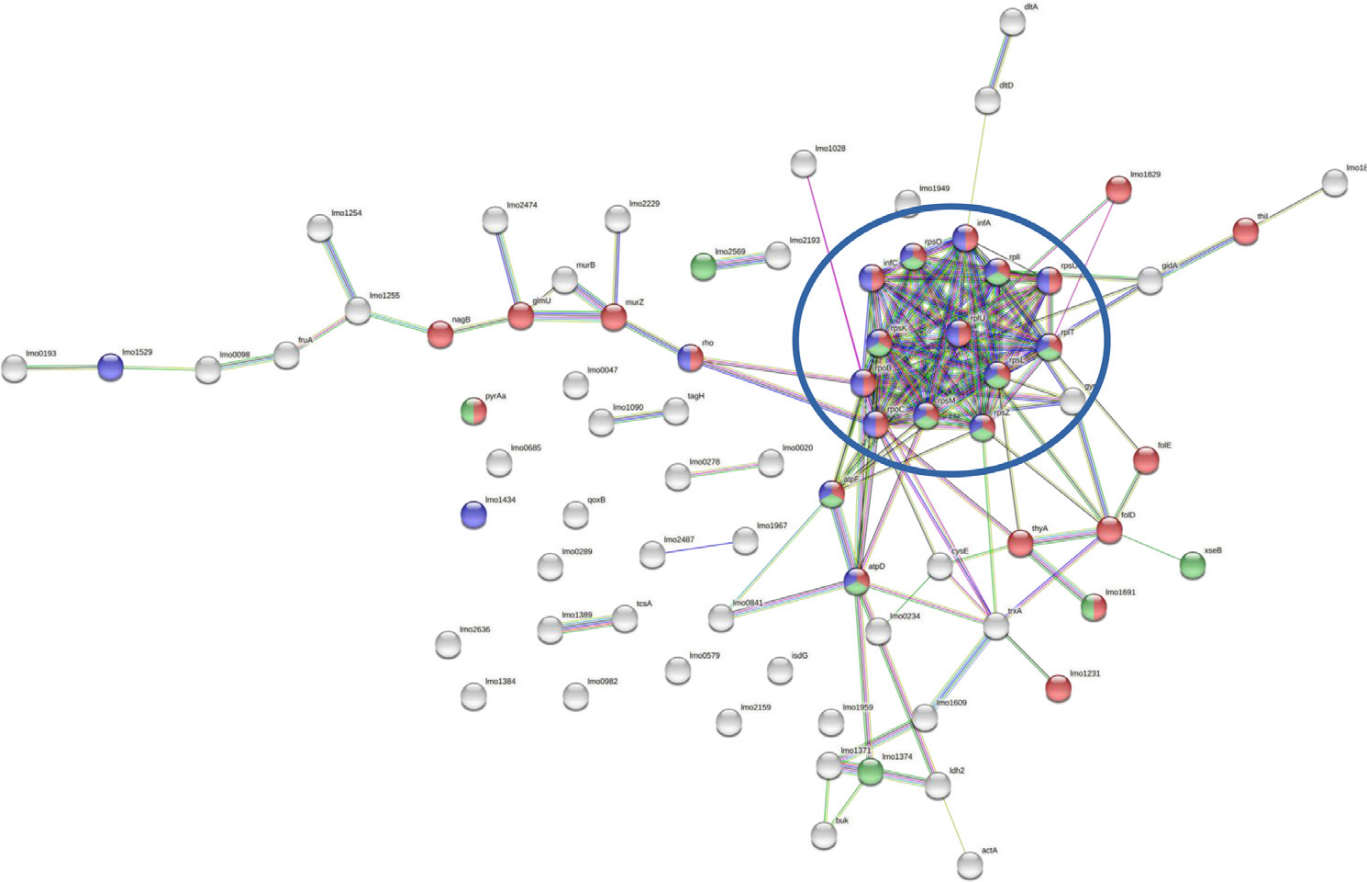
Protein-protein interaction network of upregulated *Listeria monocytogenes* ATCC 7466 proteins after interaction with nisin-loaded liposomes. The proteins are represented by nodes whereas their interactions by edges. The line colors indicate different types of know (pink and pastel blue), predicted (green, red and blue) and others (yellow, black and gray) interactions. The proteins (identified by its code gene) in red color related with cellular nitrogen compounds biosynthesis, blue color with translation and channel activity, and green color with protein-containing complex. The network was constructed with STRING v11.05.

The proteomics data analyzed in this article is related to our previous research article titled “Proteomic analysis reveals differential responses of *Listeria monocytogenes* to free and nanoencapsulated nisin” [1]. The data of this article includes the set proteins identified using UniProt, with VIP (Variable Importance in Projection) score ≥1.0, obtained from of *L. monocytogenes* ATCC 7644 cells incubated for 1 h with sublethal concentrations of either free or liposome-encapsulated nisin. The set of proteins showing upregulation as compared with the control cells are summarized in Table 1. This set of proteins denotes the global mechanism, in terms of protein expression and triggered by *L. monocytogenes* cells after treatment with free and nanoencapsulated nisin. These two groups of proteins were selected to explore the interactions among proteins that showed differential expression. An *in silico* analysis was conducted using the free available software STRING (Search Tool for the Retrieval of Interacting Genes/Proteins) version 11.05. For each set of proteins that were upregulated in response to free nisin and/or nanoencapsulated nisin, it was determined the number of protein-protein interactions documented in the database and the network functional enrichment. The complete set of proteins obtained from the STRING enrichment analysis for both free and liposome encapsulated nisin, are showed in the supplementary Table S1 and Table S2, respectively (available in the on-line repository MSV000089076). In addition, a graph linking proteins symbolized by nodes with known interactions with the encoded genes of the identified proteins was assembled for visualization purposes. Different colors were used to evaluate the functional characteristics of proteins that were present in the nodes observed for upregulated proteins in treatments with free nisin (Fig. 1) and nanoencapsulated nisin (Fig. 2). A network with 140 edges with two relevant nodes was obtained with the analysis of proteins upregulated by free nisin, including a great quantity of membrane proteins. These protein clusters present biological connection and are related to stress response mechanisms in *L. monocytogenes*. The interaction analysis of upregulated proteins by liposome-loaded nisin showed 156 edges with a single protein network, the same observed in free nisin, related to ribosomal proteins.

**Table 1:** Upregulated protein/peptide reports of *Listeria monocytogenes* ATCC 7644 treated by sub-lethal concentration of free nisin (Nis) or liposome-encapsulated nisin (LNis) for 1 h.

Uniprot accession	Gene name	Annotation	Treatment	Description
Q8YA70	*lmo0289*	*lmo0289*	Nis / LNis	Annotation not available
Q8Y828	*lmo1090*	*lmo1090*	Nis / LNis	Annotation not available
Q8Y615	*lmo1887*	*lmo1887*	Nis / LNis	Hypothetical protein; belongs to the methyltransferase superfamily
Q8Y437	*lmo2636*	*lmo2636*	Nis/ LNis	Hypothetical protein; flavin transferase that catalyzes the transfer of the FMN moiety of FAD and its covalent binding to the hydroxyl group of a threonine residue in a target flavoprotein
Q8Y7A4	*lmo1384*	*lmo1384*	LNis	Hypothetical protein; belongs to the UPF0176 family
Q8Y7C5	*lmo1360*	*folD*	LNis	Methenyltetrahydrofolate cyclohydrolase; catalyzes the oxidation of 5,10-methylenetetrahydrofolate and then the hydrolysis to 10-formyltetrahydrofolate
Q8YAC0	*lmo0226*	*folK*	Nis	6-hydroxymethyl-7,8-dihydropterin pyrophosphokinase; involved in the biosynthesis of tetrahydrofolate from GTP
Q8YA71	*lmo0288*	*lmo0288*	Nis	Annotation not available
Q8YAJ0	*lmo0135*	*lmo0135*	Nis	Annotation not available
Q8Y6B7	*lmo1774*	*purK*	Nis	Phosphoribosylaminoimidazole carboxylase ATPase subunit; catalyzes the ATP-dependent conversion of 5-aminoimidazole ribonucleotide and HCO_3_^−^ to N5-carboxyaminoimidazole ribonucleotide
Q8Y8Q4	*lmo0842*	*lmo0842*	Nis	Annotation not available
Q92CZ4	*lmo1028*	*lmo1028*	LNis	Hypothetical protein; belongs to the UPF0356 family
Q8YAR2	*lmo0053*	*rplI*	LNis	50S ribosomal protein L9; binds to the 23S rRNA
Q8Y8E7	*lmo0957*	*nagB*	LNis	Glucosamine-6-phosphate isomerase; catalyzes the reversible isomerization-deamination of glucosamine 6-phosphate to form fructose 6-phosphate and ammonium ion
Q8Y6S4	*lmo1609*	*lmo1609*	LNis	Annotation not available
P0A4L3	*lmo1233*	*trxA*	LNis	Component of the thioredoxin-thioredoxin reductase system
Q8Y626	*lmo1874*	*thyA*	LNis	Thymidylate synthase; catalyzes the reductive methylation of 2′-deoxyuridine-5′-monophosphate (dUMP) to 2′-deoxythymidine-5′-monophosphate (dTMP) while utilizing 5,10-methylenetetrahydrofolate (mTHF) as the methyl donor and reductant in the reaction, yielding dihydrofolate (DHF) as a by-product
P65110	*lmo2610*	*infA*	LNis	Translation initiation factor IF-1; one of the essential components for the initiation of protein synthesis
Q8Y7A4	*lmo1384*	*lmo1384*	LNis	Hypothetical protein; belongs to the UPF0176 family
P66383	*lmo2608*	*rpsM*	Nis / LNis	30S ribosomal protein S13; located at the top of the head of the 30S subunit, contacts several helices of the 16S rRNA
Q8Y7B5	*lmo1371*	*lmo1371*	Nis / LNis	Dihydrolipoyl dehydrogenase; E3 component of the branched-chain alpha-keto acid dehydrogenase complex; catalyzes the oxidation of dihydrolipoamide to lipoamide
Q8Y7B6	*lmo1370*	*buk*	Nis/ LNis	Butyrate kinase; belongs to the acetokinase family
P33379	*lmo0204*	*actA*	Nis/ LNis	Actin-assembly inducing protein precursor; virulence factor required for host cell microfilament interaction
P66401	*lmo2619*	*rpsZ*	Nis / LNis	30S ribosomal protein S14; binds 16S rRNA, required for the assembly of 30S particles and may also be responsible for determining the conformation of the 16S rRNA at the A site
Q48762	*lmo0234*	*lmo0234*	Nis / LNis	Hypothetical protein; RNAse
Q8Y4F7	*lmo2487*	*lmo2487*	Nis / LNis	Annotation not available
Q48754	*lmo1388*	*tcsA*	Nis / LNis	CD4+ T-cell stimulating antigen
P66352	*lmo2607*	*rpsK*	Nis / LNis	30S ribosomal protein S11; located on the platform of the 30S subunit, bridges several disparate RNA helices of the 16S rRNA
Q8Y701	*lmo1529*	*lmo1529*	Nis / LNis	Annotation not available
Q8Y6Y9	*lmo1542*	*rplU*	Nis / LNis	50S ribosomal protein L21; this protein binds to 23S rRNA in the presence of protein L20
Q8Y5V6	*lmo1949*	*lmo1949*	Nis / LNis	Hypothetical protein; belongs to the pseudouridine synthase RsuA family
Q8Y4B8	*lmo2533*	*atpF*	Nis / LNis	F_1_F_0_ ATP synthase; produces ATP from ADP in the presence of a proton or sodium gradient
Q8Y6U0	*lmo1592*	*thiI*	Nis / LNis	Thiamine biosynthesis protein ThiI; catalyzes the ATP-dependent transfer of a sulfur to tRNA to produce 4-thiouridine in position 8 of tRNAs, which functions as a near-UV photosensor
Q8YAU3	*lmo0020*	*lmo0020*	Nis / LNis	Annotation not available
Q8Y9F0	*lmo0579*	*lmo0579*	Nis / LNis	Annotation not available
Q8Y486	*lmo2569*	*lmo2569*	Nis / LNis	Annotation not available
Q8Y7L9	*lmo1255*	*lmo1255*	Nis / LNis	Annotation not available
Q8Y8C6	*lmo0982*	*lmo0982*	Nis / LNis	Annotation not available
Q8Y4U6	*lmo2335*	*fruA*	Nis / LNis	FruA protein; sugar transporter, phosphoenolpyruvate-dependent phosphotransferase system
Q7AP82	*lmo0685*	*lmo0685*	Nis / LNis	Flagellar motor protein MotA; with MotB forms the ion channels that couple flagellar rotation to proton/sodium motive force across the membrane and forms the stator elements of the rotary flagellar machine
Q8Y7A1	*lmo1389*	*lmo1389*	Nis / LNis	Annotation not available
Q8Y7P2	*lmo1231*	*lmo1231*	Nis / LNis	Annotation not available
Q8YAU9	*lmo0014*	*qoxB*	Nis / LNis	AA3-600 quinol oxidase subunit I; belongs to the heme-copper respiratory oxidase family
Q8Y670	*lmo1829*	*lmo1829*	Nis / LNis	Annotation not available
Q8Y8Q5	*lmo0841*	*lmo0841*	Nis / LNis	Calcium-transporting ATPase; catalyzes the hydrolysis of ATP coupled with the transport of calcium
Q8Y547	*lmo2229*	*lmo2229*	Nis / LNis	Annotation not available
Q8Y5U6	*lmo1959*	*lmo1959*	Nis / LNis	Annotation not available
Q8Y7M0	*lmo1254*	*lmo1254*	Nis / LNis	Annotation not available
Q9RLT9	*lmo0258*	*rpoB*	Nis / LNis	DNA-directed RNA polymerase subunit beta; DNA-dependent RNA polymerase catalyzes the transcription of DNA into RNA using the four ribonucleoside triphosphates as substrates
P0DJP1	*lmo1469*	*rpsU*	Nis / LNis	30S ribosomal protein S21; belongs to the bacterial ribosomal protein bS21 family
Q7AP78	*lmo0971*	*dltD*	Nis / LNis	DltD protein; involved in lipoteichoic acid biosynthesis phathway
Q8YA96	*lmo0259*	*rpoC*	Nis / LNis	DNA-directed RNA polymerase subunit beta; DNA-dependent RNA polymerase catalyzes the transcription of DNA into RNA using the four ribonucleoside triphosphates as substrates
Q8Y4A3	*lmo2551*	*rho*	Nis / LNis	Transcription termination factor Rho; facilitates transcription termination by a mechanism that involves Rho binding to the nascent RNA, activation of Rho's RNA-dependent ATPase activity, and release of the mRNA from the DNA template
Q8Y4A2	*lmo2552*	*murZ*	Nis / LNis	UDP-*N*-acetylglucosamine 1-carboxyvinyltransferase; cell wall formation
Q8Y8D4	*lmo0974*	*dltA*	Nis / LNis	D-alanine-poly(phosphoribitol) ligase subunit 1; catalyzes the first step in the D-alanylation of lipoteichoic acid (LTA), the activation of D-alanine and its transfer onto the D-alanyl carrier protein (Dcp) DltC
Q8YAV6	*lmo0007*	*gyrA*	Nis / LNis	Dna gyrase subunit a; type II topoisomerase that negatively supercoils closed circular double-stranded (ds) DNA in an ATP-dependent manner to modulate DNA topology and maintain chromosomes in an underwound state
Q8Y5A9	*lmo2159*	*lmo2159*	Nis / LNis	Annotation not available
Q8Y664	*lmo1836*	*pyrAa*	Nis / LNis	Carbamoyl phosphate synthase small subunit; Belongs to the CarA family
Q8YAR7	*lmo0047*	*lmo0047*	Nis / LNis	Hypothetical protein
Q8Y4G9	*lmo2474*	*lmo2474*	Nis / LNis	Hypothetical protein; displays ATPase and GTPase activities
Q92C24	*lmo1330*	*rpsO*	Nis / LNis	30S ribosomal protein S15; one of the primary rRNA binding proteins, binds directly to 16S rRNA where it helps nucleate assembly of the platform of the 30S subunit by binding and bridging several RNA helices of the 16S rRNA
P66372	*lmo2656*	*rpsL*	Nis / LNis	30S ribosomal protein S12; with S4 and S5 plays an important role in translational accuracy
P0A3L1	*lmo1785*	*infC*	Nis / LNis	Translation initiation factor if-3; IF-3 binds to the 30S ribosomal subunit and shifts the equilibrium between 70S ribosomes and their 50S and 30S subunits in favor of the free subunits, thus enhancing the availability of 30S subunits on which protein synthesis initiation begins
Q8Y776	*lmo1420*	*murB*	Nis / LNis	UDP-*N*-acetylenolpyruvoylglucosamine reductase; cell wall formation
Q92EH3	*lmo0484*	*isdG*	Nis / LNis	Heme-degrading monooxygenase IsdG; allows bacterial pathogens to use the host heme as an iron source
Q8Y6Z6	*lmo1534*	*ldh2*	Nis / LNis	L-lactate dehydrogenase; catalyzes the conversion of lactate to pyruvate
Q8YAB2	*lmo0238*	*cysE*	Nis / LNis	Serine acetyltransferase; involved in the subpathway that synthesizes L-cysteine from L- serine
Q8YAD8	*lmo0193*	*lmo0193*	Nis / LNis	Hypothetical protein
Q8YA81	*lmo0278*	*lmo0278*	Nis / LNis	Sugar ABC transporter ATP-binding protein; belongs to the ABC transporter superfamily
Q8Y843	*lmo1075*	*tagH*	Nis / LNis	Teichoic acid ABC transporter ATP-binding protein; part of the ABC transporter complex TagGH involved in teichoic acids export
Q8YAM0	*lmo0098*	*lmo0098*	Nis / LNis	Annotation not available
Q8YAD4	*lmo0198*	*glmU*	Nis / LNis	Glucosamine-1-phosphate *N*-acetyltransferase; catalyzes the last two sequential reactions in the de novo biosynthetic pathway for UDP-*N*-acetylglucosamine (UDP-GlcNAc)
Q7AP53	*lmo2193*	*lmo2193*	Nis / LNis	Peptide ABC transporter ATP-binding protein; belongs to the ABC transporter superfamily
Q8Y5T8	*lmo1967*	*lmo1967*	Nis/ LNis	Toxic ion resistance protein; belongs to the TelA family
Q8Y767	*lmo1434*	*lmo1434*	Nis / LNis	Hypothetical protein; RNase that has 5′-3′ exonuclease and possibly endonuclease activity
Q8Y7C3	*lmo1362*	*xseB*	Nis / LNis	Exodeoxyribonuclease VII small subunit; bidirectionally degrades single-stranded DNA into large acid- insoluble oligonucleotides, which are then degraded further into small acid-soluble oligonucleotides
Q8Y7B2	*lmo1374*	*lmo1374*	Nis / LNis	Annotation not available
Q8Y6J3	*lmo1691*	*lmo1691*	Nis / LNis	Deoxyuridine triphosphate nucleotidohydrolase; enzyme involved in nucleotide metabolism, produces dUMP, the immediate precursor of thymidine nucleotides and it decreases the intracellular concentration of dUTP so that uracil cannot be incorporated into DNA
Q8Y3M5	*lmo2810*	*gidA*	Nis / LNis	tRNA uridine 5-carboxymethylaminomethyl modification enzyme GidA; NAD-binding protein involved in the addition of a carboxymethylaminomethyl group at the wobble position (U34) of certain tRNAs
P66103	*lmo1783*	*rplT*	Nis / LNis	50S ribosomal protein L20; binds directly to 23S ribosomal RNA and is necessary for the in vitro assembly process of the 50S ribosomal subunit
Q8Y4C1	*lmo2529*	*atpD*	Nis / LNis	ATP synthase F_0_F_1_ subunit beta; produces ATP from ADP in the presence of a proton gradient across the membrane
Q8Y5 × 1	*lmo1933*	*folE*	Nis / LNis	GTP cyclohydrolase 1; involved in the first step of tetrahydrofolate biosynthesis; catalyzes the formation of formate and 2-amino-4-hydroxy-6-(erythro-1,2,3-trihydroxypropyl) dihydropteridine triphosphate from GTP and water; forms a homopolymer
Q8Y6Z1	*lmo1539*	*lmo1539*	Nis	Glycerol transporter; belongs to the MIP/aquaporin
Q8Y703	*lmo1527*	*secD*	Nis	Part of the Sec protein translocase complex
Q8Y980	*lmo0653*	*lmo0653*	Nis	Hypothetical protein
Q8Y839	*lmo1079*	*lmo1079*	Nis	Annotation not available
Q8Y8 × 2	*lmo0770*	*lmo0770*	Nis	Annotation not available
Q8Y8E9	*lmo0955*	*lmo0955*	Nis	Hypothetical protein
Q8Y7A4	*lmo1384*	*lmo1384*	LNis	Hypothetical protein; belongs to the UPF0176 family

## 2 Experimental Design, Materials and Methods

### 2.1 Samples

The influence of free and nanoliposome-encapsulated nisin on the proteomic profile of *L. monocytogenes* was investigated using the strain ATCC 7644 (American Type Culture Collection, Manassas, VA, USA). The bacterial strain was retrieved from the stock culture maintained in Brain Heart Infusion (BHI) broth (Kasvi, São José dos Pinhais, Paraná, Brazil) containing 20% (v/v) glycerol for long-standing storage. To acclimatize the strain to the experimental conditions, an aliquot of the culture (0.1 mL) was inoculated into 9.9 mL BHI broth and incubated overnight in a shaker at operating 37 °C and 125 rpm. Afterwards, the bacterial cells were cultivated in BHI broth for 24 h at 37 °C using a 1% (v/v) inoculum. For the analysis, cells were then cultivated at 37 °C until they reached the mid-exponential growth phase (at hour 6 and OD_600_ about 0.4). At this time, either free or liposome-encapsulated nisin were added at 0.3 µg/mL final concentration in separate treatments [1]. The liposomes were prepared by the thin film hydration method using purified phosphatidylcholine (Phospholipon 90G, provided by Lipoid, Ludwigshafen, Germany) as detailed in a previous work [2]. This method result in stable liposomes with entrapment efficiency of nisin superior to 90% [3]. Cells of *L. monocytogenes* incubated under the same conditions but without any treatment were used as control. The bacterial cells were incubated at 37 °C for 1 h, then harvested by centrifugation at 5000 *g* at 4 °C for 10 min, and the pellets were washed three times with 2 mL of PBS pH 7.4 and then reserved for protein extraction [4]. Each treatment was performed in triplicate (biological replicates). For the analysis, samples of *L. monocytogenes* treated with free and liposome-encapsulated nisin were compared with control *L. monocytogenes* cultures.

### 2.2 Protein digestion and preparation of peptides

Protein digestion was performed according to standard protocols for complex protein mixtures [5]. In summary, the protocol consisted of the following steps:

(a) Denaturation of extracts containing 100 μg of *L. monocytogenes* proteins using 8 M urea (1:1, v/v) for 30 min;
(b) Reduction of the samples using 5 mM dithiothreitol (DTT, Sigma-Aldrich, St. Louis, MO, USA) during 25 min at 56 °C;
(c) Alkylation with 14 mM iodoacetamide (IAA, Sigma-Aldrich), during 30 min at room temperature in a light protected ambient;
(d) Addition of 5 mM DTT followed by 15 min incubation to eliminate the remaining IAA;
(e) Dilution of the samples with 50 mM ammonium bicarbonate (1:5, v/v) to reach a concentration of 1.6 M urea, containing 1 mM CaCl_2_ as a trypsin cofactor;
(f) Addition of trypsin (Sequencing Grade Modified Trypsin, Promega, WI, USA), prepared at 20 μg/mL in 50 mM ammonium bicarbonate, at 1:50 E/S ratio;
(g) Incubation at 37 °C during 16 h for protein digestion;
(h) Addition of 2% (v/v) trifluoroacetic acid to stop the proteolytic reaction.

Afterwards, samples were centrifuged at 14,000 *g* for 20 min, and the supernatants were collected and applied to C18 reverse phase Stage Tips for desalination [6]. Stage Tips were previously conditioned with methanol and equilibrated with 0.1% (v/v) formic acid. Samples were loaded and 0.1% (v/v) formic acid was used to wash the salt residues. Peptides were then eluted with 60% (v/v) acetonitrile containing 0.1% (v/v) formic acid and the samples were freeze-dried and stored at −20 °C until LC-MS/MS analysis.

### 2.3 Nano-LC-MS/MS analysis

The dried peptide samples were suspended in 10 μL formic acid (0.1%, v/v). Aliquots of 3 μL were analyzed using a LTQ Orbitrap Velos mass spectrometer (Thermo Fisher Scientific, Waltham, MA, USA), coupled to EASY-nano-LC system equipped with a Proxeon nanoelectrospray ion source (Proxeon Biosystem, West Palm Beach, FL, USA). The LC-MS/MS parameters were as follows:

Column: 20 cm x 75 μm ID, 5 μm particle size PicoFrit column (New Objective, Littleton, MA, USA).
Elution conditions: 2-90% (v/v) acetonitrile gradient containing 0.1% (v/v) formic acid, eluted at a flow rate of 300 μL/min over 65 min.
Instrumental procedures: set up in the data-dependent acquisition (DDA) mode; nanoelectrospray voltage 2.2 kV; source temperature 275 °C; resolution *r* = 60,000; collision energy of 35% for CID (collision-induced dissociation) fragmentation of most abundant ions with charge ≥2, sequentially isolated to a target value of 5000; dynamic exclusion enabled at size list of 500 peptides, exclusion duration of 60 s and a repetition count of 1.

### 2.4 Data processing

Raw MS files were processed with the MaxQuant software version v1.3.0.3 [7], and the Andromeda engine was employed to match MS/MS spectra against the *Listeria monocytogenes* UniProt protein sequence database and contaminant protein sequence (https://www.uniprot.org/). The following parameters were used for MaxQuant: trypsin digestion, with maximum 2 missed cleavages and minimum peptide length of 7; cysteine carbamidomethylation as a fixed modification, while variable modifications were methionine oxidation and acetylation (Protein N-term); mass tolerance for peptides and fragments was set to ±20 ppm and ±0.1 Da; peptide and protein false discovery rate (FDR) cut-off was set to 0.01. The statistical analysis was performed using MetaboAnalyst 3.068. Only proteins with valid intensity values of label-free quantification (LFQ) detected in ≥50% of the samples were considered for analysis. The data was subjected to partial least squares discriminate statistical analysis (PLS-DA), then was established the cutoff value VIP (Variable Importance in Projection) and proteins with VIP score ≥1.0 were considered as upregulated [8]. All the proteins with VIP score ≥1.0 were characterized using UniProt and regrouped as upregulated proteins.

From the treatments, two groups of proteins (free and nanoencapsulated-encapsulated nisin) were selected for examination of the interactions among proteins showing differential expression (upregulation). An *in silico* analysis was conducted using the free available software STRING (Search Tool for the Retrieval of Interacting Genes/Proteins) database version 11.05 [9]. The number of protein-protein interactions registered in the database were determined for the proteins that were differentially over expressed. For visualization purposes, a diagram was assembled linking proteins depicted by nodes with recognized connections with the identified proteins. At the same time, proteins with common gene ontology terms were identified by different colors.

## Ethics Statements

This work does not involve human subjects, animal experiments or data collected from social media platforms.

## CRediT Author Statement

**Cristian Mauricio Barreto Pinilla:** Methodology, Software, Writing; **Paolo Stincone:** Methodology, Data curation, Writing; **Adriano Brandelli:** Conceptualization, Writing, Editing.

## Declaration of Competing Interest

The authors declare that they have no known competing financial interests or personal relationships that could have appeared to influence the work reported in this paper.

## Data Availability

MSV000089076 (Original data) (MASSIVE).
